# The clinical and radiological outcomes of multilevel minimally invasive transforaminal lumbar interbody fusion

**DOI:** 10.1007/s00586-012-2619-y

**Published:** 2012-12-19

**Authors:** Sang-Hyuk Min, Jae-Sung Yoo

**Affiliations:** 1 Department of Orthopaedic Surgery, Dankook University College of Medicine, 16-5 Anseo-dong, Cheonan, 330-715 Korea; 2 Department of Preventive Medicine, Dankook University College of Medicine, 16-5 Anseo-dong, Cheonan, 330-715 Korea

**Keywords:** Multilevel interbody fusion, Minimally invasive surgery, Transforaminal, Lumbar interbody fusion

## Abstract

**Purpose:**

To investigate the clinical and radiological outcomes of multilevel minimally invasive transforaminal lumbar interbody fusion (MITLIF) in multilevel degenerative lumbar diseases.

**Methods:**

Of 172 patients who could be followed-up for at least 1 year after undergoing a MITLIF, a total of 127 patients in whom unilateral cages were used through a unilateral approach (consisting of 69 patients for single-level, 40 for two-level, and 18 for three- or higher-level) were retrospectively studied as subjects. In this study, clinical assessment parameters included Visual Analog Scale (VAS) score and Oswestry Disability Index (ODI), while radiologic assessment parameters included disc height, segmental lordotic angle, and lumbar lordotic angle. At the last follow-up, the level of bone fusion was determined in accordance with the Brantigan and Steffee criteria for classification of fusion results.

**Results:**

The VAS scores of back pain and radiating leg pain tended to improve postoperatively, and showed no significant difference among groups (*p* > 0.05). In terms of ODI, the results of functional assessments also indicated no significant difference among groups (*p* > 0.05). Similarly, there was no statistically significant difference in disc height, segmental lordotic angle, lumbar lordotic angle, and bone fusion depending on the number of fusion levels (*p* > 0.05).

**Conclusions:**

Regardless of the number of fused levels, satisfactory clinical and radiological outcomes of MITLIF were seen in patients with spinal stenosis, which suggests that the said surgical procedure may be useful even for patients with multilevel spinal stenosis.

## Introduction

With recent increases in average life expectancy and degenerative diseases attributed to population aging, the incidence of multilevel degenerative lumbar diseases is rising, and the frequency of multilevel lumbar fusion is also getting higher. Although interbody fusion via conventional posterior approach is widely used to treat multilevel degenerative lumbar diseases, it is suggested that this surgical procedure is associated with higher incidence of several complications resulting from severer muscle damage and blood loss due to the dissection and retraction of broad muscular and soft tissues required for wide laminectomy, pedicle screw placement, and securing fusion beds [1–5]. In some recent studies, it was reported that minimally invasive transforaminal lumbar interbody fusion (MITLIF) minimized blood loss and soft tissue damage, reduced postoperative complications and low back pains and consequently contributed significantly to shortening the length of hospital stay and recovery time, suggesting satisfactory clinical and radiological outcomes [6–10].

However, many surgeons still believe that multilevel MITLIF has some limitation in providing adequate neural decompression, is difficult to correct the sagittal plane with poly-axial screws, and furthermore, causes heavier correction loss. They also think that higher incidence of fusion failure as a result of insufficient bone grafting leads to poor or unsatisfactory clinical and radiological outcomes. The results of studies concerning single-level MITLIF have been reported, whereas the results of studies addressing fully clinical and radiological outcomes of multilevel MITLIF have not yet been published.

In this context, we attempted to investigate the clinical and radiological outcomes of multilevel MITLIF in the manner of comparatively analyzing the outcomes of MITLIF depending on the number of fusion levels.

## Materials and methods

### Subjects

Of 172 patients with spinal stenosis who underwent a MITLIF for spinal stenosis in our hospital between March 2006 and January 2010 and were followed-up for at least 1 year postoperatively, 127 patients in whom unilateral cages were used through unilateral approach were enrolled as subjects of this study and retrospectively studied. The subjects consisted of 47 men and 80 women and their mean age was 56.78 years (30–82 years). The mean follow-up period was 24.53 months (18–52 months), and the exclusion criteria for subject selection included undergoing previously a spinal surgery, receiving neural decompression at any other levels simultaneously with interbody fusion, and the presence of the unbalanced sagittal plane and coronal plane as complicated by spondylolisthesis or degenerative scoliosis. For the number of fusion levels, 69 subjects corresponded to single level, 40 subjects to two levels, and 18 subjects to three or more levels (consisting of 10 to three levels, 6 to four levels, and 2 to five levels) (Table 1).

**Table 1:** Patient’s data

	Overall	One level (*n* = 69)	Two level (*n* = 40)	Three levels or more (*n* = 18)
Age (years)	56.78 (30–82)	54.10 (30–82)	59.87 (32–77)	61.21 (48–74)
M:F	45:78	29:40	12:28	6:12
Follow-up (months)	24.53 (18–52)	25.24 (18–48)	23.46 (18–52)	21.53 (18–38)
BMI	24.72 (14.63–43.51)	24.63 (14.63–32.51)	24.76 (19.05–43.51)	25.08 (19.05–33.02)
BMD (Hip)	−0.84 (−3.58 to 1.50)	−0.60 (−3.58 to 1.50)	−1.07 (−3.30 to 1.50)	−1.40 (−2.90 to −0.30)

### Surgical procedures and techniques

**Fig. 1:**
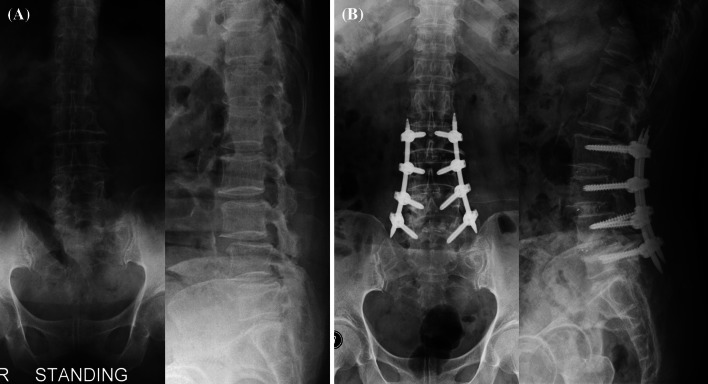
Multilevel MI-TLIF procedure. **a** Preoperative radiography; **b** postoperative radiography

A 2.5-cm long incision was made at a site distant from approximately 2.5 cm from the center, the multifidus muscle was separated from the longissimus muscle to get access between the two muscles, the laminae and facet joints were reached with microlumbar retractor, the upper halves of the inferior and superior articular processes were removed under surgical operating microscope, the ligamentum flavum was removed to expose the dura and the nerve root running around and out of the upper pedicle, and finally, discectomy was performed. Next, a shaver was employed to elongate sequentially the interbody intervals, and the superior and inferior lumbar endplates to be fused were curetted to make preparations. For patients with spinal stenosis, the patients were inclined to the opposite side to the surgeon, and then a high-speed orthopedic drill was used under surgical operating microscope to perform a unilateral sublaminar decompression and contralateral neural decompression. After checking that adequate neural decompression has been given, the interbody space was filled up with the bone chips obtained during laminectomy and either autogenous bone chips harvested from the posterior superior iliac crest or synthetic hydroxyapatite, subsequently a cage filled with autogenous bone chips was inserted, and percutaneous pedicle screw fixation was conducted (Fig. 1).

### Analytical procedures

The patients undergoing a MITLIF were assigned, depending on the number of fused levels, to three groups: single-level group (69 subjects), two-level group (40 subjects), and three- or higher-level group (18 subjects) to analyze comparatively different groups. Their gender, age, past history (blood pressure, diabetes, and endocrine disease), body height, body weight, body mass index (BMI), ASA score, bone mineral density (BMD), and diagnosis were investigated.

For clinical and radiological assessments, postoperative outcomes were comparatively analyzed on the basis of measurements made preoperatively, immediately postoperatively, and at the final follow-up, and immediate postoperative assessments were conducted when the patient could stand up during hospital stay (at day 3 on average). Clinical assessment parameters included Visual Analog Scale (VAS) score and Oswestry Disability index (ODI), while radiological assessment parameters included preoperative and postoperative radiological findings (disc height, segmental lordotic angle in the sagittal plane), radiological fusion rate at the final follow-up, and the correction loss between postoperative outcomes and final follow-up outcomes, based upon simple standing radiographs of Picture Archiving Communication System (PACS). The means of measurements made twice by each of an orthopedist majoring in spinal orthopedics and a radiologist majoring in musculoskeletal system were used for analyses and assessments.

Regarding disc height measurements, disc height was defined as an interbody height measured within the perpendicular line passing a median point between the line passing the anterior superior part of the lower lumbar vertebral body and the line passing the posterior inferior part of the upper lumbar vertebral body among lines perpendicular to the line going along the superior margin of the lumbar vertebral body. In multilevel MITLIF group, the means of disc height measurements at respective levels were regarded as representative values, followed by comparative analysis.

**Fig. 2:**
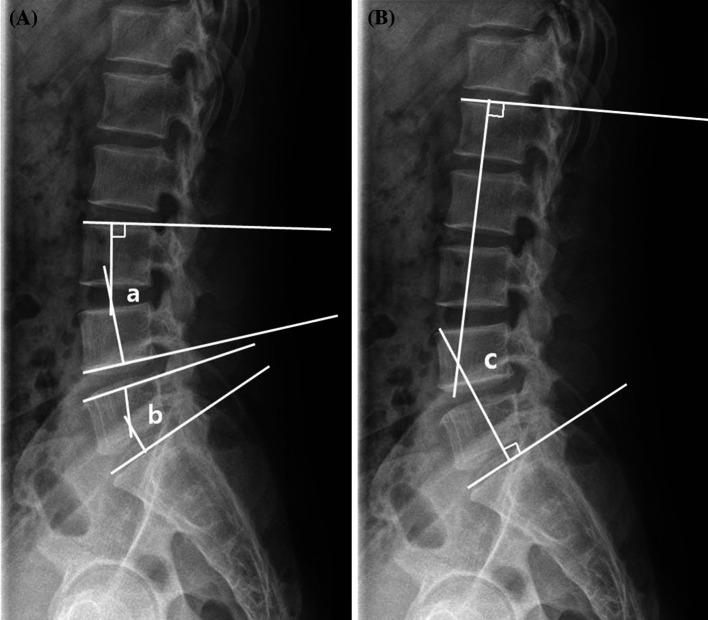
Cobb’s angle for segmental lordosis and whole lumbar lordosis. **A** The segmental lordosis at L3–4 (*a*) was defined as the angle subtended by the superior endplate line of L3 and the inferior endplate line of L4. The segmental lordosis at L5–S1 (*b*) was defined as the angle subtended by the superior endplate line of L5 and superior endplate line of S1. **B** The whole lumbar lordosis (*c*) was defined as the angle subtended by the superior endplate line of L1 and superior endplate line of S1

Segmental lordotic angle was defined as an acute angle made by the respective perpendicular lines to the line passing the superior margin of the upper lumbar vertebral body and the line passing the inferior margin of the lower lumbar vertebral body at the fused level, and lumbar lordotic angle was defined as an acute angle made by the respective perpendicular lines to the line passing the superior margin of lumbar vertebra 1 and the line passing the superior margin of sacrum 1 (Fig. 2). Since segmental and lumbar lordotic angles vary with the number of fusion levels, any differences among measurements were comparatively analyzed to determine the levels of postoperative correction and of correction loss at the final follow-up. Radiological determinations of fusion were based on simple radiographs taken at the final follow-up and were assessed according to the Brantigan and Steffee criteria for classification of fusion results (Table 2).

**Table 2:** Classification of fusion results (Brantigan and Steffee 1991)

	Overall
A	Obvious collapse of construct due to pseudoarthrosis, loss of disc height, vertebral slip, broken screws, displacement of the cage, resorption of bone graft
B	Probable significant resorption of the bone graft due to pseudoarthrosis, major lucency, or gap visible in fusion area (2 mm around the entire periphery of graft)
C	Uncertain non-union, bone graft visible in the fusion area at approximately the density originally achieved at surgery. A small lucency or gap may be visible involving a portion of the fusion area with at least half of the flat area
D	Probable fusion bone bridges entire fusion area with at least the density achieved at surgery. There should be no lucency between the door and vertebral bone. Fusion bone in the fusion area is radiographically more dense and mature than originally achieved by surgery
E	Optimally, there is no interface between the donor and vertebral bone, although a sclerotic line between the graft and vertebral bone indicates fusion. Other signs of the solid fusion include mature bony trabeculae bridging the fusion area, resorption of the anterior traction spur, anterior progression of the graft within disc space, and fusion of facet joints

### Statistic analysis

For statistics, Window SPSS (version 19.0) was used to perform one-way ANOVA analyses; the age, gender, past history, BMI, and BMD of the three groups were adjusted, and bone mineral density (BMD) and *p* value <0.05 were considered to indicate statistically significant difference.

## Results

### Clinical outcomes and assessments

Mean VAS scores of back pain were 6.25 points preoperatively, 1.92 points postoperatively, and 0.85 points at the final follow-up: in single-level fusion group, 6.06 points preoperatively, 1.85 points postoperatively, and 0.73 points at the final follow-up; in two-level fusion group, 6.63 points preoperatively, 2.00 points postoperatively, and 0.97 points at the final follow-up; and, in three- or higher-level fusion group, 6.14 points preoperatively, 2.07 points postoperatively, and 1.07 points at the final follow-up, which suggests that there is no significant difference among the groups (*p* > 0.05) (Table 3).

**Table 3:** Clinical results

	Overall	One level (*n* = 69)	Two level (*n* = 40)	Three levels or more (*n* = 18)
VAS (back pain)				
Pre-operation	6.25 ± 0.50	6.06 ± 0.65	6.63 ± 0.95	6.14 ± 1.68
Post-operation	1.92 ± 0.30	1.85 ± 0.40	2.00 ± 0.61	2.07 ± 0.73
Final follow-up	0.850 ± 0.22	0.73 ± 0.29	0.97 ± 0.45	1.07 ± 0.53
VAS (leg pain)				
Pre-operation	7.73 ± 0.38	7.66 ± 0.52	7.85 ± 0.71	7.71 ± 1.05
Post-operation	1.76 ± 0.76	1.60 ± 0.49	1.90 ± 0.62	2.14 ± 1.10
Final follow-up	0.85 ± 0.25	0.62 ± 0.25	1.27 ± 0.62	0.78 ± 0.61
ODI				
Pre-operation	24.70 ± 1.78	23.49 ± 2.33	26.42 ± 3.37	25.78 ± 4.22
Post-operation	11.09 ± 1.19	9.68 ± 1.41	12.85 ± 2.48	13.07 ± 3.11
Final follow-up	8.37 ± 1.15	7.40 ± 1.46	9.40 ± 2.07	10.21 ± 4.56

Mean VAS scores of leg pain were 7.73 points preoperatively, 1.76 points at week 2 postoperatively, and 0.85 points at the final follow-up, suggesting postoperative improvements: in single-level fusion group, 7.66 points preoperatively, 1.60 points postoperatively, and 0.62 points at the final follow-up; in two-level fusion group, 7.85 points preoperatively, 1.90 points postoperatively, and 1.27 points at the final follow-up; and, in three- or higher-level fusion group, 7.71 points preoperatively, 2.14 points postoperatively, and 0.78 points at the final follow-up, which suggests no statistically significant difference among the groups (*p* > 0.05) (Table 3).

Mean ODI scores were 24.70 points preoperatively, 11.09 points at week 2 postoperatively, and 8.47 points at the final follow-up, suggesting postoperative improvements: in single-level fusion group, 23.49 points preoperatively, 9.68 points postoperatively, and 7.40 points at the final follow-up; in two-level fusion group, 26.42 points preoperatively, 12.85 points postoperatively, and 9.40 points at the final follow-up; and, in three- or higher-level fusion group, 25.78 points preoperatively, 12.85 points postoperatively, and 10.12 points at the final follow-up, which suggests no statistically significant difference among the groups (*p* > 0.05) (Table 3).

### Radiological outcomes and assessments

For those receiving a multilevel fusion, the means of disc height measurements at respective levels were regarded as representative values and comparatively analyzed. Mean disc heights were 9.17 mm preoperatively, 11.98 mm postoperatively, and 10.81 mm at the final follow-up: in single-level fusion group, 9.60 mm preoperatively, 12.3 mm postoperatively, and 10.9 mm at the final follow-up; in two-level fusion group, 8.53 mm preoperatively, 11.41 mm postoperatively, and 10.61 mm at the final follow-up; and, in three- or higher-level fusion group, 8.90 mm preoperatively, 11.76 mm postoperatively, 10.96 mm at the final follow-up, which suggests that there is no statistically significant difference among the groups (Table 4).

**Table 4:** Radiological findings

	Overall	One level (*n* = 69)	Two level (*n* = 40)	Three levels or more (*n* = 18)
Disc height (mm)				
Pre-operation	9.17 ± 0.46	9.60 ± 0.61	8.52 ± 0.78	8.90 ± 1.45
Post-operation	11.98 ± 0.43	12.36 ± 0.58	11.41 ± 0.73	11.76 ± 1.37
Final follow-up	10.81 ± 0.40	10.90 ± 0.56	10.61 ± 0.65	10.96 ± 1.38
Segmental lordotic angle (°)				
Pre-operation	15.22 ± 1.83	11.57 ± 2.01	18.82 ± 3.49	22.92 ± 5.43
Post-operation	19.37 ± 1.44	16.20 ± 1.54	22.50 ± 2.68	26.07 ± 4.14
Final follow-up	17.49 ± 1.35	14.50 ± 1.46	20.60 ± 2.49	23.35 ± 4.02
Lumbar lordotic angle (°)				
Pre-operation	34.14 ± 2.23	35.43 ± 3.97	32.22 ± 3.89	34.14 ± 8.24
Post-operation	42.65 ± 1.18	44.13 ± 2.26	40.47 ± 3.74	41.64 ± 7.45
Final follow-up	36.69 ± 2.03	42.05 ± 2.12	36.25 ± 4.03	37.92 ± 7.74

As lordotic angle measurements were dependent on different fused levels and thus it is impossible to compare them in an objective manner, comparative analyses were conducted in terms of difference between preoperative and postoperative measurements, difference between postoperative and final follow-up measurements, and difference between preoperative and final follow-up measurements.

Mean segmental lordotic angles were 15.22° preoperatively, 19.37° postoperatively, and 17.49° at the final follow-up with 4.14° of difference between preoperative and postoperative measurements, 1.87° of difference between postoperative and final follow-up measurements, and 2.27° of difference between preoperative and final follow-up measurements: specifically, in single-level fusion group, 4.62° of difference between preoperative and postoperative measurements, 1.69° of difference between postoperative and final follow-up measurements, and 2.92° of difference between preoperative and final follow-up measurements; in two-level fusion group, 3.67° of difference between preoperative and postoperative measurements, 1.90° of difference between postoperative and final follow-up measurements, and 1.77° of difference between preoperative and final follow-up measurements; and, in three- or higher-level fusion group, 3.14° of difference between preoperative and postoperative measurements, 2.71° of difference between postoperative and final follow-up measurements, and 0.42° of difference between preoperative and final follow-up measurements, which suggests no statistically significant difference among the groups (*p* > 0.05; Table 4).

Mean lumbar lordotic angles were 34.24° preoperatively, 42.65° postoperatively, and 39.69° at the final follow-up with 8.41° of difference between preoperative and postoperative measurements, 2.95° of difference between postoperative and final follow-up measurements, and 5.45° of difference between preoperative and final follow-up measurements: specifically, in single-level fusion group, 8.69° of difference between preoperative and postoperative measurements, 2.07° of difference between postoperative and final follow-up measurements, and 6.62° of difference between preoperative and final follow-up measurements; in two-level fusion group, 8.25° of difference between preoperative and postoperative measurements, 4.22° of difference between postoperative and final follow-up measurements, and 4.02° of difference between preoperative and final follow-up measurements; and, in three- or higher-level fusion group, 7.50° of difference between preoperative and postoperative measurements, 3.71° of difference between postoperative and final follow-up measurements, and 3.78° of difference between preoperative and final follow-up measurements, which suggests no statistically significant difference among the groups (*p* > 0.05; Table 4).

Regarding bone fusion at the final follow-up, successful bone fusion was achieved in 114 of 127 cases (89.96 %): specifically, 62 of 69 cases (89.85 %) in single-level fusion group; 36 of 40 cases (90.00 %) in two-level fusion group; and 16 of 18 cases (88.89 %) in three- or higher-level fusion group, which suggests no statistically significant difference among the groups (*p* > 0.05).

## Discussion

It has been reported that posterior fusion or posterior interbody fusion with conventional pedicle screws has the problems of increased intraoperative blood loss and thus higher incidence of perioperative complications and prolonged recovery time which are attributed to excessive retraction involving soft tissue damage and surrounding muscle pain due to its approach manner [1–5, 11]. On the other hand, since MITLIF is designed to minimize damage to soft tissues and surrounding muscles by separating the multifidus muscle from the longissimus muscle and getting access to the space between the two muscles, and intended to reduce excessive retraction during interbody fusion in the manner of approaching the disc via the outside of the foramen, it has the advantages of lessening muscle damage associated with surgical approach and reducing postoperative blood loss [6–10, 12, 13].

In addition, satisfactory clinical outcomes of single-level MITLIF have been reported. Potter et al. [14] reported that satisfactory clinical outcomes were shown in 80 % of 100 patients who underwent a single MITLIF and were followed-up over 2 years. Foley et al. [15] reported improvements in ODI score from 55 points preoperatively to 11 points postoperatively. The authors investigated VAS and ODI scores and analyzed them comparatively depending on the number of fused levels. Mean VAS score decreased from 7.73 points preoperatively to 0.85 points at the final follow-up, mean ODI score also decreased from 24.7 points preoperatively to 8.37 points at the final follow-up, and the comparative analyses of the scores depending on the number of fused levels did not show significant difference. This suggests that multilevel MITLIF may improve satisfactorily the relevant symptoms and enable patients to achieve their functional restoration.

Meanwhile, radiological outcomes of MITLIF have also been reported. Kim et al. [8] reported that interbody height, segmental lordotic angle, and lumbar lordotic angle increased significantly at the final follow-up compared to preoperative values, and 95.4 % of bone fusion rate was observed. Similarly, in this study, interbody height, segmental lordotic angle, and lumbar lordotic angle at the final follow-up increased significantly from preoperative values, with 96.04 % (118 of 123 cases) of bone fusion rate, and the comparative analyses of them depending on the number of fused levels showed no statistically significant difference (*p* > 0.05). This suggests that multilevel MITLIF may help to achieve satisfactory radiological reduction.

Foley et al. [15] suggest that compared to conventional surgery, MITLIF is more difficult to detect surgical landmarks during operation because the operating area or space is narrow and limited, it requires three-dimensional understanding of the spinal structures and nerves, and it takes surgeons a longer time to learn the surgical techniques and procedures because they should familiarize themselves with use and manipulations of surgical operating microscope and surgical instruments. Also in this study, mean operation time was 154.43 min in single-level group, 202.35 min in two-level group and 237.14 min in three- or higher-level group, indicating longer operation time required for MITLIF than that for conventional fusion. This is probably due to longer time required for neural decompression and percutaneous screw placement and combination through skin incisions made at multiple levels each. In this light, there have been various attempts to shorten the operation time of MITLIF, and Min and Lee [16] and Min and Hwang [17] reported the possibility to decrease statistically significantly the operation time by unilateral approach, compared with bilateral approach. Furthermore, it is thought that the operation time can shorten as the surgeon gets more familiar with the surgical procedures and techniques of MITLIF. Similarly, in this study, of 58 subjects receiving a multilevel MITLIF until January 2010, mean operation time was 225.19 min in 29 subjects undergoing the operation in the first half of the period, while it was 185.37 min in the other 29 subjects undergoing the operation in the latter half, which probably suggests that the operation time can shorten as the surgeon gets increasingly familiar with the surgical procedures and techniques.

**Fig. 3:**
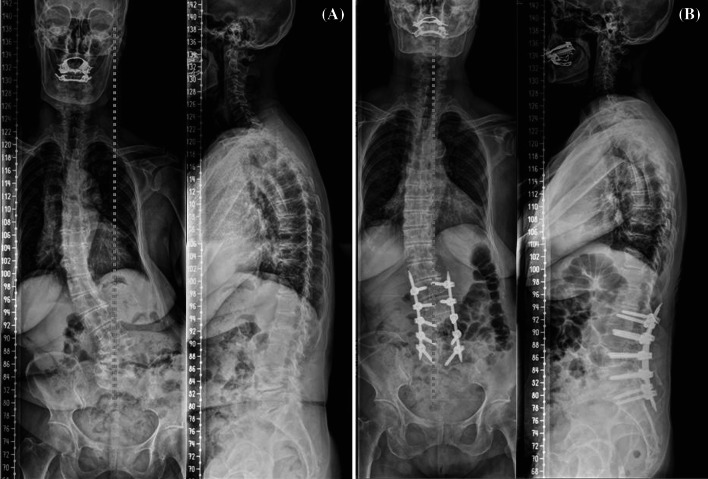
Multilevel MI-TLIF procedure. **a** Preoperative radiography; **b** postoperative radiography showing reduction of scoliotic curvature

**Fig. 4:**
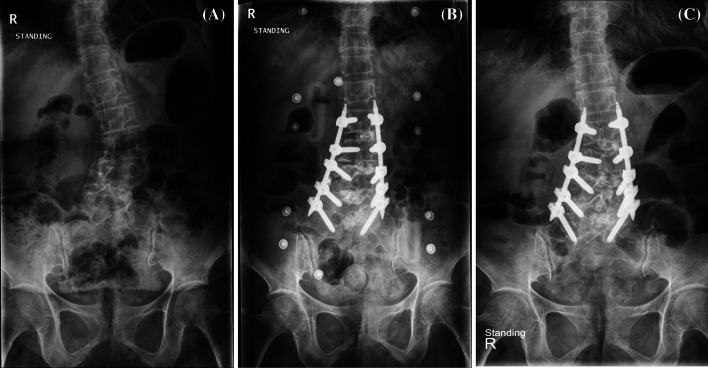
Multilevel MI-TLIF procedure. **a** Preoperative radiography; **b** postoperative radiography showing reduction of scoliotic curvature; **c** last follow-up radiography showing loss of reduction

There were some cases excluded from the subject population of this study due to the presence of degenerative scoliosis as comorbidity. In the cases, the authors performed decompression and deformity correction using percutaneous pedicle screw fixation simultaneously with multilevel MITLIF, with the intention of minimizing intraoperative blood loss in consideration for their general conditions. In a case of them, Cobb angle in single curve was corrected from 44° preoperatively to 10° postoperatively and 16° at the final follow-up (at year 2 postoperatively) on the radiographs (Fig. 3), and VAS score of leg pain and ODI score at the final follow-up were 1 and 12 points, respectively, and improved, indicating the limited levels of radiologic correction but satisfactory clinical outcomes. In another case with degenerative scoliosis as comorbidity, VAS score of leg pain and ODI score at the final follow-up were 2 and 11 points, respectively, suggesting satisfactory outcomes; however, Cobb angle improved from 34° preoperatively to 8° postoperatively and 18° at the final follow-up (at year 1 postoperatively) indicating remarkable correction loss (Fig. 4). It is considered that constant studies of correction level and loss in a larger sample size of degenerative scoliosis patients are necessary to clarify the therapeutic effects of multilevel MITLIF in patients with degenerative scoliosis as comorbidity.

This study has the following limitations: first, because of a small number of the subjects included in three- or higher-level group and the different numbers of fused levels, it was impossible to compare interbody height, segmental lordotic angle, and lumbar lordotic angle measurements under same conditions; second, as this study was designed to analyze comparatively MITLIF cases depending on the number of fused levels, it was not compared with conventional posterior fusion or posterior interbody fusion using conventional pedicle screws. To overcome these limitations and obtain more objective results, it may be required to perform further comparative studies in a larger number of multilevel MITLIF cases versus conventional multilevel posterior fusion or posterior interbody fusion. Nevertheless, it is considered this study is meaningful as the first study attempted to analyze comparatively the clinical and radiological outcomes of multilevel MITLIF depending on the number of fused levels.

## Conclusions

Regardless of the number of fused levels, minimally invasive transforaminal lumbar interbody fusion showed satisfactory clinical and radiological outcomes in patients with spinal stenosis, which suggests that it may be useful even for patients with multilevel spinal stenosis.
